# A profile of the Centre for Health Record Linkage

**DOI:** 10.23889/ijpds.v4i2.1142

**Published:** 2019-11-29

**Authors:** K Irvine, R Hall, L Taylor

**Affiliations:** 1 NSW Health, St Leonards NSW 2065 Australia; 2 Choicemaker LLC, Princeton New Jersey 08540 USA

## Abstract

**Context:**

The Centre for Health Record Linkage (CHeReL) was established in 2006 as a dedicated health and human services data linkage facility for two Australian jurisdictions, New South Wales and the geographically-nested Australian Capital Territory. The two jurisdictions have their own Governments and separate Health and Human Service systems.

**Purpose and Operations:**

The primary purpose of the CHeReL is to make linked administrative and routinely collected health data available to researchers and government within relevant regulatory and governance frameworks. The CHeReL’s data governance and technical operations draw on international best practice and have been refined by learnings from other data linkage centres.

**Outcomes:**

Over twelve years of operation, more than 2,320 unique investigators from 140 institutions have used the CHeReL, producing 615 publications in peer-reviewed literature. A robust pipeline of new development is expected to further amplify the use of linked data for cutting edge medical research and support a vision of data-informed policy and data-driven government services.

## Background

In response to increasing evidence of the utility of linked data at a population level for health research, such as the Oxford Record Linkage Study [1] and the work of the Manitoba Centre for Health Policy [2, 3], the then NSW Department of Health established a record linkage service in 1994 to support health research and management of health services. In 2005, following marked increases in the demand for the data linkage service, the Sax Institute commissioned the Data Linkage Australia Partners to evaluate the case for a data linkage facility in NSW and to recommend a preferred model based on international best practice and the views of stakeholders in NSW. In 2006, eight organisations agreed to contribute funding for the first three years of operation of the CHeReL: NSW Department of Health, ACT Health, Cancer Institute NSW, Clinical Excellence Commission, the University of Newcastle, University of New South Wales, University of Sydney and Sax Institute.

The governance, funding and operation of the CHeReL during the establishment phase was based on the recommendations by Data Linkage Australia, informed by systematic investigation of international best practice, experiences in Western Australia and interviews with 44 principal stakeholders in NSW and the ACT [4]. The features of relatively well developed systems in 2005 were reviewed, including the Oxford Record Linkage Study, Scottish Record Linkage System, Rochester Epidemiology Project, Manitoba Population Health Information System, British Columbia Linked Health Database and Western Australia data linkage system. Key success factors or best practice included the development of strong collaborations with government data custodians [5], efforts to streamline the authorising environment [5, 6], appropriate engagement of stakeholders including consumers [5], the efficiency of comprehensive linkage systems relative to ad hoc servicing of project specific linkage requests [7, 8] and implementation of the separation principle to protect privacy [6].

While many original features from the establishment phase endure, further development of the governance, funding, operating models and technology over time has been critical to support growth and diversification of the CHeReL user base and responsiveness to time- critical health system priorities.

This paper describes the current approach of the CHeReL and reflects on the evolution of the data linkage operating model. Organisationally, the CHeReL is a business unit of the NSW Health, Health System Support Group and managed and primarily funded by the NSW Ministry of Health. The CHeReL is also supported by the Population Health Research Network which is an initiative of the Australian Government being conducted as part of the National Collaborative Research Infrastructure Strategy.

## Approach

### Population setting

The primary population base for the CHeReL includes both New South Wales and the Australian Capital Territory, Australia, although records from all Australian jurisdictions are held and used. The estimated resident population of NSW and the ACT at the end of September 2018 was 8.4 million, representing approximately 33.6% of the Australian population [9].

### Operating models

A project by project linkage service enables external datasets to be linked to each other or to a comprehensive system of enduring person and family-based links. External datasets are linked to the comprehensive system at a point in time for projects and are not automatically included within the system. The comprehensive linkage system contains a centralised repository of linked personal identifiers. Enduring links are stored in perpetuity in the CHeReL Master Linkage Key so that records do not need to be repeatedly matched for different studies [7], timeframes for accessing linked data are reduced [10] and linkage quality for new datasets with limited identifiers is improved through leveraging pre-linked arrays of personal identifiers for an individual [11, 12].

Personal information for data linkage is separated from content information (clinical or service data), consistent with the internationally accepted implementations of the separation principle. Prior to 2017, the CHeReL did not handle content information and relied exclusively on a distributed data linkage or classical linkage model [13,14,15] formally recommended as best practice in Australia during the establishment of the CHeReL in 2006 [4]. Under this model, the CHeReL used personal identifiers to link and create anonymous ‘linkage keys’ that were passed to data custodians. ‘Linkage keys’ were attached to approved clinical or service data by custodians and provided to researchers, who could merge data together using the linkage key. While this provided a strong separation model for confidentiality [13], it had numerous well documented disadvantages [15] and in 2014 a centralised content data delivery capability and repository of unlinked content data was shown to produce comparatively faster and more predictable timeframes [15].

**Figure 1: Multiple operating models with the separation of data and functions:**
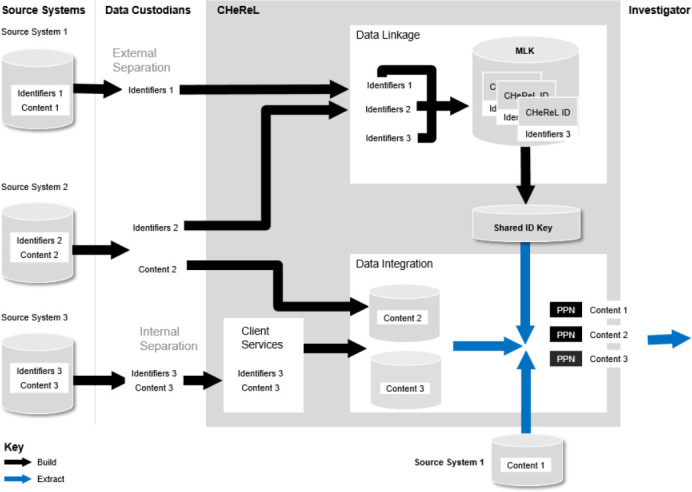


Since 2017 the CHeReL handles content information in a separate Data Integration Unit (DIU) and offered multiple operating models to data custodians. Custodians may use the CHeReL for data linkage only or choose the CHeReL to assemble and release linked project specific extracts of content data on their behalf. The DIU may access custodian content data under a federated model or store content data within the DIU. Figure 1 below illustrates the flow of information when content data is stored within the DIU (Source Systems 2 and 3) or accessed under a federated model (Source System 1). In either case, content data remains unlinked until the point of extract when anonymous linkage keys are added for approved projects or the data is integrated by the CHeReL for quality assurance purposes. A secure process for passing linkage keys between the Data Linkage and Data Integration Units provides for detailed checking of linkage quality and refinement of linkage algorithms in a way that is not possible with third-party de-identified linked databanks [8].

Custodians may choose from three models, shown as Source Systems 1-3. The main difference between the models is the point of which identifiers and content will be separated prior to data linkage and where the unlinked content data is stored prior to integration. The choice of models does not impact upon the data that is integrated.

Typically, data custodians split files into two, personal identifiers and content information, before provision to the CHeReL. The information flow is shown in Figure 1 above as Source System 2. However, following consultation with other data linkage units in Canada and Australia, an internal separation function has been offered. Under this model the separate Client Services Unit will split files on behalf of custodians, followed by destruction of original files where data custodians are unwilling or unable to perform this function themselves.

The development of current operating models was overseen by a committee comprising representatives from NSW and ACT government health agencies. The committee considered high level principles, consultation processes, data governance, privacy and security. Staff of the established WA Data Linkage Branch and PopData BC provided detailed information of their operating models to assist the Committee. Further consultations were carried out with the CHeReL’s Community Advisory Committee, key data custodians and research users. Use of the models in association with the Master Linkage Key were approved by the NSW Population and Health Services Research Ethics Committee and the ACT Health Human Research Ethics Committee.

### Architecture and information technology

The CHeReL operates on a SQL Server 2017 platform (SQL Enterprise Edition) running on VMware vSphere environment. Servers are accessed through dual Netscaler Access Gateways to Citrix Xenapp servers where applications are published. Storage is on a HP EVA SAN used exclusively by the CHeReL.

### Governance, legislation and management

The CHeReL is a business unit of NSW Health and under the governance and management of NSW Health. Data governance arrangements include legal, ethical, policy and procedural elements.

In NSW, a legal basis is required for use of personally identifying information for record linkage. The CHeReL is bound by the *Health Records Information and Privacy Act 2002* for personal health information and the *Privacy and Personal Information Protection Act 1998* for personal information. As a NSW Health Agency, the CHeReL also has obligations under the *Health Administration Act 1982* and the *Public Health Act 2010* in relation to collection and disclosure of information.

The legal framework is further supported by a range of NSW Health policies and procedures. Of particular relevance is the NSW Privacy Manual for Health Information that provides operational guidance to the legislative obligations imposed by the HRIP Act and policies on disclosure of data for research, management of health services and electronic information security.

Where linked data are used for research, and the data may identify an individual, Human Research Ethics Committee (HREC) approval is required. Where linked data are used for the funding, planning, management or evaluation of health services, linkage may be carried out with HREC approval or under the Public Health and Disease Register Provisions of the Public Health Act 2010. Under NSW privacy law, the CHeReL may provide a deduplication service for individual datasets to support data quality assurance. Approval by the Aboriginal Health and Medical Research Council Ethics Committee is required for certain projects involving Aboriginal people (http://www.ahmrc.org.au/).

### Consent model

NSW Privacy law permits record linkage with consent, and also recognises that a consent model is not always possible or practical. Other than with consent, handling of personal information for data linkage can occur for a directly related secondary purpose, such as shared medical care arrangements; or under exemptions, such as research exemptions, whereby a HREC may grant a waiver for the requirement of individual consent for use and disclosure of personal information.

### Privacy by design

Multiple privacy design strategies are used to minimise risk to personal privacy:

- separating identifier and content data and separating data linkage and data integration processes minimises collection and use in accordance with privacy principles;
- maintaining an Access Control Policy that restricts staff access to specific data required for their role;
- ensuring that data releases contain approved variables and a unique project specific person number;
- ensuring that data release agreements stipulate conditions on end users including privacy protections and security arrangements
- ensuring robust information security

The CHeReL maintains an ISO 27001 aligned Information Security Management System (ISMS) that is independently audited and aligns to Australian Government requirements including: the Australian Signals Directorate Information Security Manual, the Australian Government’s Protective Security Policy Framework and the Australian Cyber Security Centre Essential Eight.

### Data linkage

Both deterministic and probabilistic techniques are used for data linkage, however probabilistic methods are favoured for enhancing linkage quality with longitudinal administrative data that may be characterised by errors and changes over time [14]. Personal information is typically used for probabilistic linkage although approximate matching of encoded personal information is also carried out.

Where full personal identifiers are available for linkage, the CHeReL uses ChoiceMaker software (https://www.choicemaker.com) for probabilistic linkage [16]. ChoiceMaker record matching is distinguished from classical probabilistic approaches such as Felligi-Sunter by automated blocking [17, 18] and maximum entropy modelling. ChoiceMaker features an extensible plugin architecture that allows the incorporation of custom and third-party software libraries for data standardisation, name and address parsing, and data validation. The system also allows users to make use of stacked data (for example multiple addresses for a person) and provides for user-specified action to group records (for example solving transitive linkage problems where record A is a high probability match to both record B and C but record B and C are low probability matches to each other).

Automated blocking makes linkages highly repeatable regardless of operator experience and simple to configure while maximum entropy modelling makes them highly accurate. Another advantage of maximum entropy modelling is it allows CHeReL to efficiently choose the training data used for weight computations.

Like the Felligi-Sunter or Naive Bayes techniques used by other record matching systems, CHeReL uses a set of simple Boolean tests, called features in the machine learning literature, that evaluate the similarity between fields across records to assess whether two records represent the same person. The relative significance of each test is computed by regression, or training, against a collection of record pairs, each of which has been reviewed and classified by data experts as a “match” or a “differ”. Unlike Felligi-Sunter or Naive Bayes, maximum entropy models do not assume or require conditional independence of the similarity tests [19]. Similar to Felligi-Sunter, match probabilities are converted into computed linkage decisions using classification against upper and lower thresholds. Thresholds are manually set for individual projects to optimise linkage quality or to minimise clerical review if required for the specific project. For enduring links in the Master Linkage Key, thresholds are set to optimise linkage quality.

Internal model validation, national benchmarking using published approaches [20] and published research studies evidence good linkage quality [21]. High quality linkage has also been achieved using near real-time techniques however incomplete enumeration of the most recently occurring events in jurisdictional data systems impacts data quality [22].

The CHeReL has also implemented privacy preserving linkage using the Bloom filter method and approximate linkage via LinXmart initially to support an expansion of primary care data linkage in NSW [12]. Within Australia, evaluations of this method have shown high quality linkage on large-scale, real world health datasets [23, 12], particularly where linkage is configured to leverage a pre-linked array of (encoded) personal identifiers over time for an individual available within an enduring linkage system [12].

### Data Sources

The CHeReL has linked over 200 distinct datasets on request under the project by project linkage model. Data has been sourced from hospitals, government agencies, non-government organisations, research institutes and private sector providers across health and other sectors.

Datasets held within the comprehensive linkage system, the Master Linkage Key, are described publicly at http://www.cherel.org.au/master-linkage-key and listed in Table 1. The time series and frequency of update for individual datasets is negotiated with respective data custodians.

The comprehensive linkage system does not include administrative health datasets relating to physician contacts or dispensing of subsidised medicines that are owned and managed by the Commonwealth government. Data for NSW/ACT residents may be linked to Commonwealth government health data for research purposes through collaboration with the Australian Institute of Health and Welfare data linkage unit on a project by project basis. Such collaboration between Australian data linkage centres, supported by the Population Health Research Network, has ensured that a broader range of whole population health and health-related data can be linked across jurisdictional systems and boundaries and made available to investigators for approved projects.

**Table 1: Characteristics of data included in the CHeReL Master Linkage Key at April 2019.** 

Dataset and Jurisdiction	Initial time period	No. of unit records	Coverage	Frequency of update

RBDM Birth registrations (NSW)	1994-2017	2,236,775 registrations	All births registered in NSW including the baby and parents	Annual
Perinatal Data Collection (NSW)	1994-2017	2,206,042 births	All births in NSW public and private hospitals including homebirths	Annual
Central Cancer Registry (NSW)	1972-2015	1,222,609 registrations	All incident cases of cancer in NSW	Annual
Notifiable Conditions Information Management System (NSW)	1993-2017	1,126,477 notifications	All notifications of certain infectious diseases and adverse events following immunisation in NSW as required under the Public Health Act 2010	Annual
Pap Test Registry (NSW)	96/97-16/17	15,075,884 tests	Cervical cancer screening test results for women residing in NSW at the time of test	Variable
BreastScreen NSW	1998-2016	6,122,103 screens	All public breast screening mammography services for women aged 40 years and over in NSW	Variable
45 and Up Study (NSW)	n/a	266,887 participants	A 10% sample of the NSW population aged 45 and over at recruitment	n/a
Ambulance NSW	2005-2018	9,888,755 cases	All emergency and non-emergency cases responded to by NSW Ambulance	Quarterly
Admitted Patient Data Collection (NSW)	2001-2018	46,137,250 episodes of care	All inpatient separated episodes of care (discharges, transfers and deaths) from all NSW public and private hospitals	Six weekly (public) six monthly (private)
Emergency Department Data Collection (NSW)	2005-2018	33,266,009 presentations	Presentations to emergency departments of public hospitals in NSW.	Six weekly
Mental Health Ambulatory Data Collection (NSW)	2001-Jun 2018	41,391,832 contacts	All care provided by NSW Health specialist mental health services for people who are not inpatients of mental health units at the time of care	Six monthly
Perinatal Death Review Database (NSW)	1994-2015	11,876 death reviews	All death reviews on around 90-95% of perinatal deaths occurring each year in NSW	Variable
RBDM death registrations (NSW)	1985-2018	1,571,752 registrations	All deaths registered in NSW	Six weekly
Cause of Death Unit Record File (NSW)	1985-2016	1,418,979 registrations	All deaths registered in NSW	Annual
BDM Birth registrations (ACT)	1997-Jun 2017	39,017 births	All births registered in ACT including the baby and parents	Annual
Perinatal Data Collection (ACT)	1997-2016	73,967 births	All births in Canberra Hospital, Calvary Public and homebirths	Annual
Kindergarten Health Check (ACT)	14/15-15/16	15,124 children’s health checks	Children enrolled in the first year of full time school in ACT) with parental consent for the health checks	Annual
Cancer Registry (ACT)	1994-2015	31,478 registrations	All cases of incident cancer diagnosed in ACT residents except basal cell carcinomas and squamous cell carcinomas.	Annual
Notifiable Diseases Management System (ACT)	2000-2015	49,501 notifications	All notifications of certain infectious diseases and conditions in NSW as required under the Public Health Act 1997	Annual
Cervical Screening Registry (ACT)	1994-2015	821,528 tests	Cervical cancer screening test results for women residing in ACT at the time of test	Variable
Admitted Patient Collection (ACT)	04/05-16/17	1,217,458 separations	Inpatient separations (discharges, transfers and deaths) from Canberra and Calvary Hospital	Annual
Emergency Department Data Collection (ACT)	05/06-16/17	1,387,223 presentations	All presentations to emergency departments of public hospitals in ACT	Annual
RBDM death registrations (ACT))	1997-Jun 2017	39,017 registrations	All deaths registered in ACT	Annual
Australian Early Development Census (Australia)	2009, 2012, 2015	859,521 children	Over 96% of children in their first year of full-time school in Australia	Three yearly
Australian and New Zealand Dialysis and Transplant Registry (Australia and New Zealand)	1963-2016	681,317 patients	Patients with end stage kidney disease receiving dialysis or renal replacement therapy in Australia and New Zealand	Annual

### Data Access

The procedures for authorisation of a data linkage project depend upon the lawful basis for data linkage, which in turn depends on the nature and purpose of the request.

Research requests and those relying on the ‘research exemptions’ in NSW privacy law require a feasibility assessment by the CHeReL and the approval of relevant data custodians and HRECs. There are a range of other lawful bases for data linkage in NSW, under the *Health Records Information and Privacy Act 2002, Public Health Act 2010 and Health Administration Regulation 2015*; in these cases, formal authorisation occurs alongside relevant data custodian approval.

Regardless of the legal basis for data linkage, governance procedures for all projects include agreements that stipulate a range of conditions on end users including privacy protections and security arrangements. Research governance arrangements have been strengthened by NSW Ministry of Health commissioned reviews of user compliance with the conditions under which the linked data was provided. These reviews were carried out in 2017 and 2019.

Bespoke extracts of approved datasets/variables with project-specific Person (and/or Family) Numbers are created for each project. Internationally, some centres apply robust techniques to achieve ‘anonymised’ extracts without “identifiable” information [24]. Within NSW, regulatory guidance notes that ‘anonymisation’ in research contexts may not equate with de-identification in privacy contexts [25]. As a consequence, authorisation and release processes comply with privacy law and principles. Variables may be restricted or treated in accordance with authorisations, however research utility is maximised through access to potentially re-identifiable data where reasonably necessary for research and with risk well managed.

Access to data may be provided through secure remote access environments, for example the Secure Unified Research Environment. Data may also be provided externally to researchers via secure ftp where the security of the proposed arrangement is assessed as satisfactory by all relevant data custodians and HRECs.

A public register of data linkage projects is available on the CHeReL website. The register includes information on the project purpose, data sources, the lead investigator and their organisation.

### Noteworthy outputs

**Figure 2: Number of records (e.g. hospital episodes of care, cancer registrations, and death registrations) released annually by the CHeReL:**
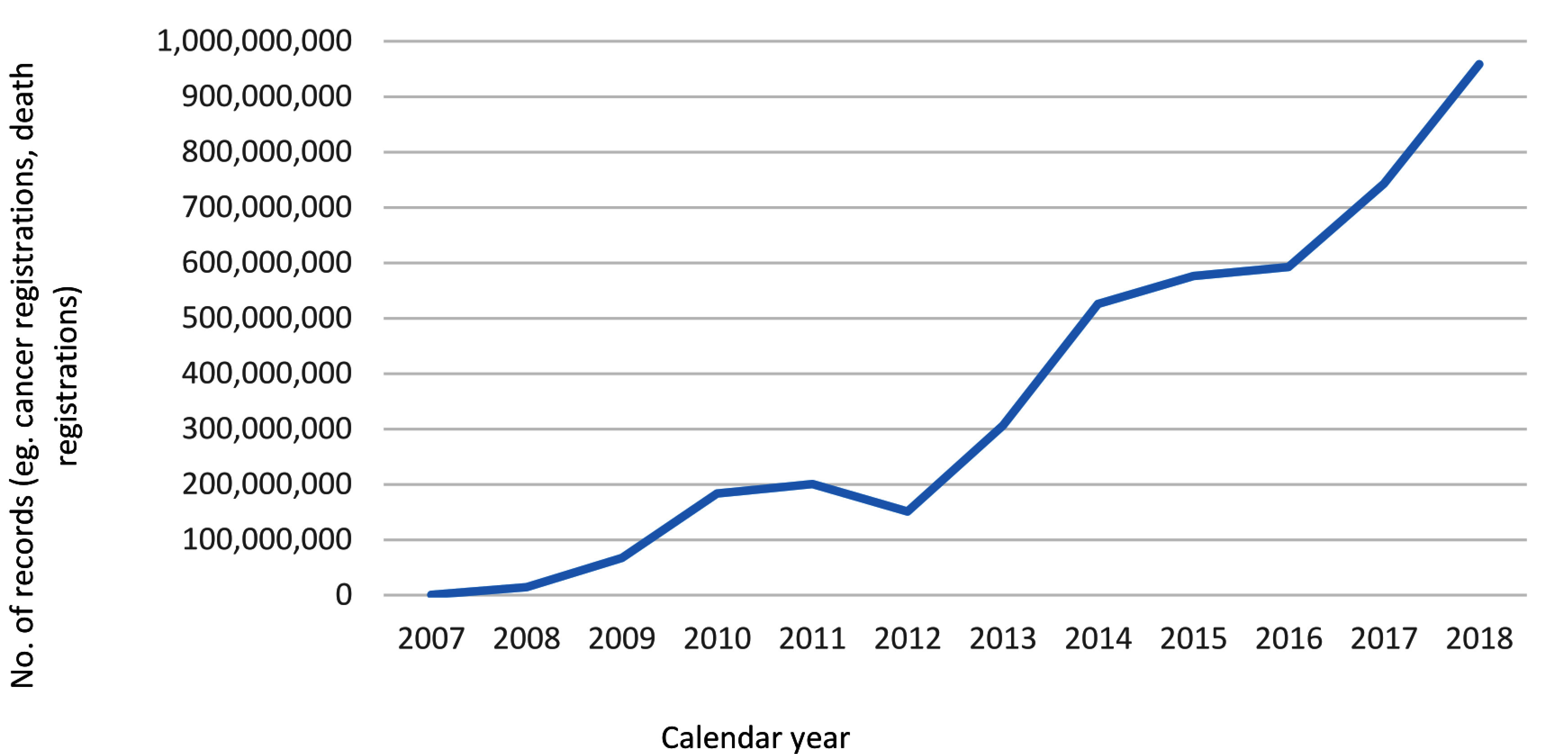


Over twelve years, more than 2,320 unique investigators from 140 institutions have been named investigators on applications to the CHeReL and hundreds more within the NSW Health system may access CHeReL linked unit record data within Public Health and Disease Registers. The number of records (e.g. hospital episodes of care, cancer registrations, death registrations) released annually for research and policy has increased steadily over time (Figure 1). For Figure 2 records are reported as the basic counting unit within each dataset (e.g. a single hospital episode of care is counted as one record) rather than the number of data points or rows of relational data that might comprise the single episode of care. Publications have also increased over time and 615 publications using data linked by the CHeReL have been identified in peer reviewed literature. Fewer than 25 peer reviewed publications were identified per annum over the first four years of full operation (2008-2011). From 2017 over 90 peer reviewed publications per annum have been identified. An updated publication list is available at http://www.cherel.org.au/publications.

Two case studies are shown below that are not currently described in the peer reviewed literature. They illustrate how law reform in NSW has strengthened the use of linked data by NSW Health and how data linkage is now being used in NSW to drive efficiency and representativeness in patient recruitment to genomic studies.

### Example 1. Law reform to enable time-critical linked data analysis in NSW Health

Linked data projects within NSW Health have traditionally been authorised under ‘research exemptions’ in privacy law with HREC approval. More recently, changes to the *Public Health Act 2010* have provided an alternate legal basis to more responsively authorise the creation of linked data assets for a range of public health purposes such as planning and evaluation of health services.

A Public Health or Disease Register, authorised under Sections 97 and 98 of the *Public Health Act 2010* can only include identifying information with consent. However, the Secretary, or a person authorised by the Secretary, may provide personal information about a person to a health records linkage organisation for the purpose of establishing and providing a unique identifier number to be used for the purposes of a register. The CHeReL has been approved as a health record linkage organisation under Section 98 of the *Public Health Act*.

Thirteen registers have been created covering areas such as communicable diseases, cancer, chronic disease, maternal and child health, and drug and alcohol use. Data are regularly linked and updated by the CHeReL. Importantly, changes to the *Public Health Act* allow data linkage to be rapidly authorised and services of the CHeReL used to inform time-critical advice to the NSW Minister for Health and NSW government. There were 113 outputs of analyses from Public Health Registers that were used to support projects or advice to government in the period 2014-15 to 2017-18.

### Example 2. Data linkage for patient recruitment studies

Data linkage can support the efficient implementation of many study designs including novel patient recruitment studies. By enabling sub-cohorts (or specific types) of patients to be identified for further testing or recruitment, use of linked data can reduce research costs, accelerate recruitment into research studies and improve cohort representativeness.

The Sax Institute’s 45 and Up Study, the largest cohort study of healthy ageing in Australia comprising more than 267,000 people provides a framework for sub-studies in which more detailed data collection or an intervention can be carried out [26]. Cohort data, combined with linked datasets from the CHeReL has been used previously to more efficiently target 45 and Up participants for recruitment into sub-studies [27]. A collaborative study currently in progress, led by Dr Jan Fullerton from Neuroscience Australia will be one of the first in Australia to use health record linkage to increase sample sizes for genomic studies in psychiatric care. In the first phase of the study, the CHeReL linked ten years of administrative health data across seven administrative datasets to enable the identification of people in the Sax Institute’s 45 and Up Study that may have bipolar disorder. These individuals will be invited to participate in the second phase of the project, which will recruit 1200 people to collect blood samples for whole genome sequencing. Using data linkage to enhance patient recruitment for this study provides a larger and more representative study population than previous studies, which were limited to recruiting patients who were actively engaged with a clinical service for treatment.

## Discussion

As a multi-stakeholder data linkage centre for two Australian jurisdictions, the CHeReL has a governance and technical infrastructure that can accommodate a broad range of custodian or investigator requirements. While flexibility benefits stakeholders, complexity comes with communication and operational challenges. Balancing operational efficiency and elegance with flexibility for stakeholders is expected to remain a focus as the CHeReL continues to diversify and grow.

The last ten years have been characterised by innovation in governance, infrastructure, methods and business process. Rapid authorisation of data linkage requests, unprecedented scale and speed of human service linkage driven by government policy priorities and the evolution of Australia’s first national data linkage network has necessitated rapid development of governance arrangements and technical infrastructure.

Future developments, in partnership with our key collaborators include:

- more frequent updating of data collections in the comprehensive linkage system and near real-time linkage to support time-critical analytics and health protection activities
- development of new linkage services featuring streamlined access to a broader array of clinical data for the NSW Health Statewide Biobank, the first and largest of its kind in the Southern Hemisphere with large-scale robotic technology to store millions of bio-specimens
- significant scale up of primary care data linkage in collaboration with the NSW Ministry of Health and Primary Health Networks
- systematisation of human services linkage and ‘de-identification’ of free text information, following on from unprecedented cross agency collaborations on data linkage for major NSW government policy priorities
- policy, operational and technical change to maximise efficiency and outcomes across Australia’s national data linkage network including short term activity to support the reduction of unnecessary duplication of HREC review for cross-jurisdictional data linkage projects

With a decade of experience in infrastructure growth and a favourable environment, the CHeReL is well placed for rapidly accelerating change.

## Conclusions

The volume of linked data from NSW and the ACT made accessible for research and policy purposes continues to increase. New developments in clinical, primary care, and cross-sectoral linkage are anticipated to further amplify the use of linked data for vital health and medical research and underpin the development and implementation of data-informed and evidence-informed policy priorities within government.
